# Delirium associated with trifluridine/tipiracil in an elderly patient with metastatic colorectal cancer: A case report

**DOI:** 10.1002/pcn5.70273

**Published:** 2025-12-15

**Authors:** Kyohei Otani, Tomohiro Kinoshita, Ryota Shindo, Naoki Shibuya

**Affiliations:** ^1^ Department of Psychiatry Kakogawa Central City Hospital Kakogawa Hyogo Japan; ^2^ Department of Surgery Kakogawa Central City Hospital Kakogawa Hyogo Japan

## Abstract

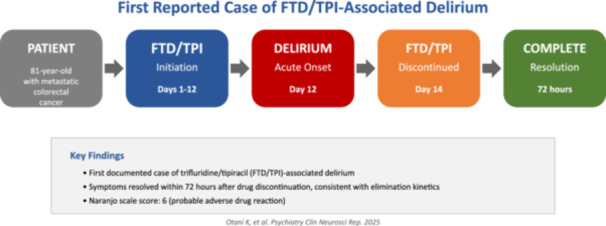

Delirium is a frequent neuropsychiatric complication in oncology, particularly among older adults receiving complex treatments. Drug‐induced delirium is frequently underrecognized despite being reversible and clinically important. Early identification and management are essential for optimizing outcomes.

Trifluridine/tipiracil (FTD/TPI, TAS‐102; Lonsurf®) is an oral nucleoside analog combining trifluridine with tipiracil, which enhances trifluridine bioavailability. It is widely used for refractory metastatic colorectal cancer, with myelosuppression and gastrointestinal events most commonly reported.^1^, ^2^ Neuropsychiatric adverse effects have rarely been described, and no prior FTD/TPI‐associated delirium reports exist.

We report a case of acute delirium temporally associated with FTD/TPI, in which consultation‐liaison psychiatry played a key role.

An 81‐year‐old man with Stage IVa rectal cancer underwent laparoscopic abdominoperineal resection. After progression through CAPOX and FOLFIRI with bevacizumab, positron emission tomography (PET)/computed tomography (CT) demonstrated further progression with lung and para‐aortic lymph node metastases, prompting fourth‐line FTD/TPI (70 mg/day on Days 1–5 and 8–12 of 28‐day cycles) plus bevacizumab.

On Day 12 of FTD/TPI therapy, he developed acute disorientation, agitation, severe insomnia, visual hallucinations, spatial misidentification, and nighttime wandering. He had no psychiatric history, and his analgesic regimen (acetaminophen and tramadol) had remained unchanged for months.

Delirium was diagnosed using DSM‐5 criteria. The patient met all required criteria: (A) disturbance in attention and awareness, (B) acute onset with fluctuating course, (C) additional cognitive disturbances including hallucinations, (D) not better explained by a preexisting neurocognitive disorder, and (E) physiological etiology related to FTD/TPI.

Neurological examination revealed fluctuating attention and perceptual disturbances without focal deficits. Cognitive screening showed preserved global cognition (Mini‐Mental State Examination 26/30, Revised Hasegawa Dementia Scale 28/30), but marked attentional impairment. Serial 7s was impaired (100 → 93 → 86 → 73, skipping 79; 2/5 points), and he was unable to complete a four‐digit backward span. Bedside observation revealed marked distractibility, frequent attention shifts, and difficulty maintaining focus. The clock drawing test was not administered.

Laboratory findings, including electrolytes, liver and renal function, and inflammatory markers, were within normal limits with no evidence of infection. Brain magnetic resonance imaging (MRI) revealed age‐related atrophy with chronic ischemic changes but no acute infarction, hemorrhage, or metastasis. Follow‐up CT (Day 38) showed stable disease without new metastases. Metabolic, infectious, and structural causes were systematically excluded.

On Day 14, a psychiatric consultation was requested. Following assessment confirming delirium, FTD/TPI was discontinued. Perospirone 4 mg daily was initiated, with risperidone 0.5 mg available (not used). Despite perospirone, symptoms persisted on Days 15–16. Marked improvement began ~24 h after FTD/TPI discontinuation, with complete resolution by Day 18 (72 h post‐discontinuation). Perospirone was discontinued after 7 days without recurrence (Figure 1).

**Figure 1:**
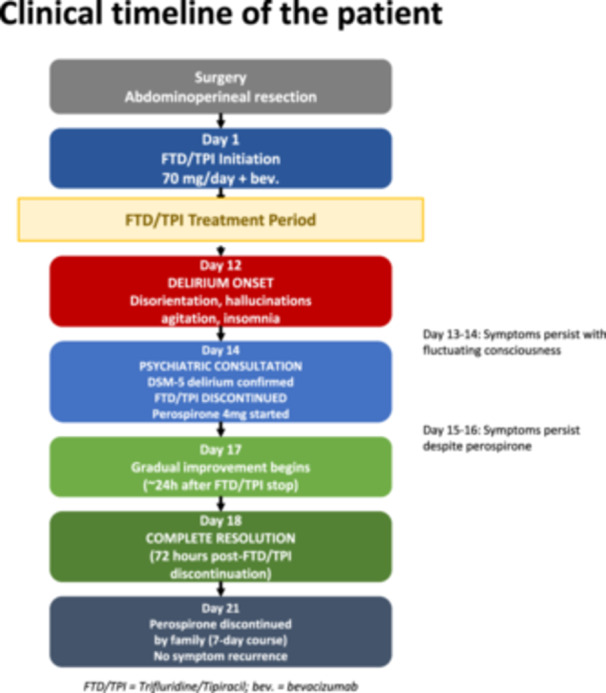
Clinical timeline of the patient. Delirium developed on Day 12 of trifluridine/tipiracil (FTD/TPI) plus bevacizumab therapy. On Day 14, a psychiatric consultation confirmed delirium using DSM‐5 criteria; FTD/TPI was discontinued, and perospirone 4 mg daily was initiated simultaneously. Despite antipsychotic treatment, symptoms persisted with minimal improvement on Days 15–16. Gradual improvement began approximately 24 h after FTD/TPI discontinuation, with complete resolution by Day 18 (72 h post‐discontinuation). Perospirone was successfully discontinued after 7 days without symptom recurrence, supporting FTD/TPI as the causative agent rather than spontaneous resolution or antipsychotic efficacy.

The temporal association, systematic exclusion of alternative causes, and positive dechallenge strongly implicated FTD/TPI as the causative agent. Naranjo Adverse Drug Reaction Probability Scale assessment yielded a score of 6, indicating a “probable” adverse drug reaction.

Delirium is multifactorial, but drug‐induced delirium should always be considered in older patients receiving polypharmacy and intensive oncological treatments. Well‐established risk factors include advanced age, multiple medications, metabolic vulnerability, sleep–wake disruption, and brain atrophy.^3^ All were present in this patient.

To distinguish improvement following FTD/TPI discontinuation from potential delayed effects of perospirone, we examined the temporal course of symptom resolution and pharmacokinetics. In this case, symptom improvement began approximately 24 h after discontinuation and resolved completely within 72 h. In contrast, the clinical effects of perospirone, an antipsychotic agent, generally require 3–5 days to manifest, which is inconsistent with the rapid improvement observed. Additionally, the half‐lives of trifluridine and tipiracil are 1.4 and 2.1 h, respectively, and the drugs would be almost completely eliminated within 72 h. This temporal course is consistent with the dechallenge effect of FTD/TPI discontinuation, supporting causality. Although UGT1A1*28 heterozygosity suggests metabolic vulnerability, its clinical significance remains speculative and requires cautious interpretation.

The mechanism remains unclear but may involve direct CNS effects, chemotherapy‐induced metabolic or inflammatory changes, or vulnerability in an atrophic brain. Hallucinations and perceptual disturbances are recognized delirium manifestations in cancer populations^4^, ^5^ but have not previously been linked to FTD/TPI.

This case illustrates that FTD/TPI should be considered in new‐onset delirium when other causes are excluded, that liaison psychiatry is crucial for diagnosis and management, and that recognition may prevent unnecessary investigations or inappropriate treatment escalation.

A limitation is the absence of standardized tools such as the Confusion Assessment Method or the Delirium Rating Scale–Revised‐98, which would have strengthened diagnostic validity and symptom quantification.

Given the increasing global use of FTD/TPI as a later‐line therapy for metastatic colorectal cancer, clinicians should maintain awareness of this rare but reversible neuropsychiatric complication. Reporting such events to pharmacovigilance systems will be essential to improve understanding and optimize patient care.

## AUTHOR CONTRIBUTIONS

All authors contributed to the conception and design of the study. Kyohei Otani conceptualized the study and drafted the manuscript. Ryota Shindo and Tomohiro Kinoshita contributed to data interpretation and manuscript revision. All authors approved the final version.

## CONFLICT OF INTEREST STATEMENT

The authors declare no conflicts of interest.

## ETHICS APPROVAL STATEMENT

This study was conducted in accordance with the principles of the Declaration of Helsinki.

## PATIENT CONSENT STATEMENT

Written consent for case reports was obtained from the patient.

## CLINICAL TRIAL REGISTRATION

N/A.

## AI AND WRITING ASSISTANCE

Claude was used to assist with grammar checking. All content, analysis, and conclusions remain the responsibility of the author.

## Data Availability

De‐identified data are available from the corresponding author on reasonable request and with institutional permission.
